# P-147. Clinical Outcomes of Typhlitis: A Large Single Center Case Series

**DOI:** 10.1093/ofid/ofaf695.373

**Published:** 2026-01-11

**Authors:** Dorothy X Kenny, Thalia McCann, Taylor Boske, Tatsiana Savenka, George R Thompson, Natascha M Tuznik, Hien Nguyen, Brian Jonas, Derek J Bays

**Affiliations:** UC Davis Medical Center, Sacramento, CA; UCHealth University of Colorado Hospital, Aurora, Colorado; UC Davis School of Medicine, Sacramento, California; UC Davis Medical Center, Sacramento, CA; University of California, Davis Medical Center, Sacramento, California; U of California Davis, Sacramento, California; VA Northern California/University of California Davis, Sacramento, California; UC Davis Health, Sacramento, California; UC Davis Health, Sacramento, California

## Abstract

**Background:**

Typhlitis, or neutropenic enterocolitis, is a life-threatening infection that occurs in neutropenic patients, particularly those with hematologic malignancies receiving chemotherapy. While typhlitis is hypothesized to arise from intestinal mucosal injury and microbial translocation, its pathogenesis is not fully understood. The available evidence consists predominantly of reviews compiling case reports. We performed a descriptive case series of a relatively large cohort while applying novel scoring systems to characterize neutropenia and antibiotic exposure.

**Table 1:**
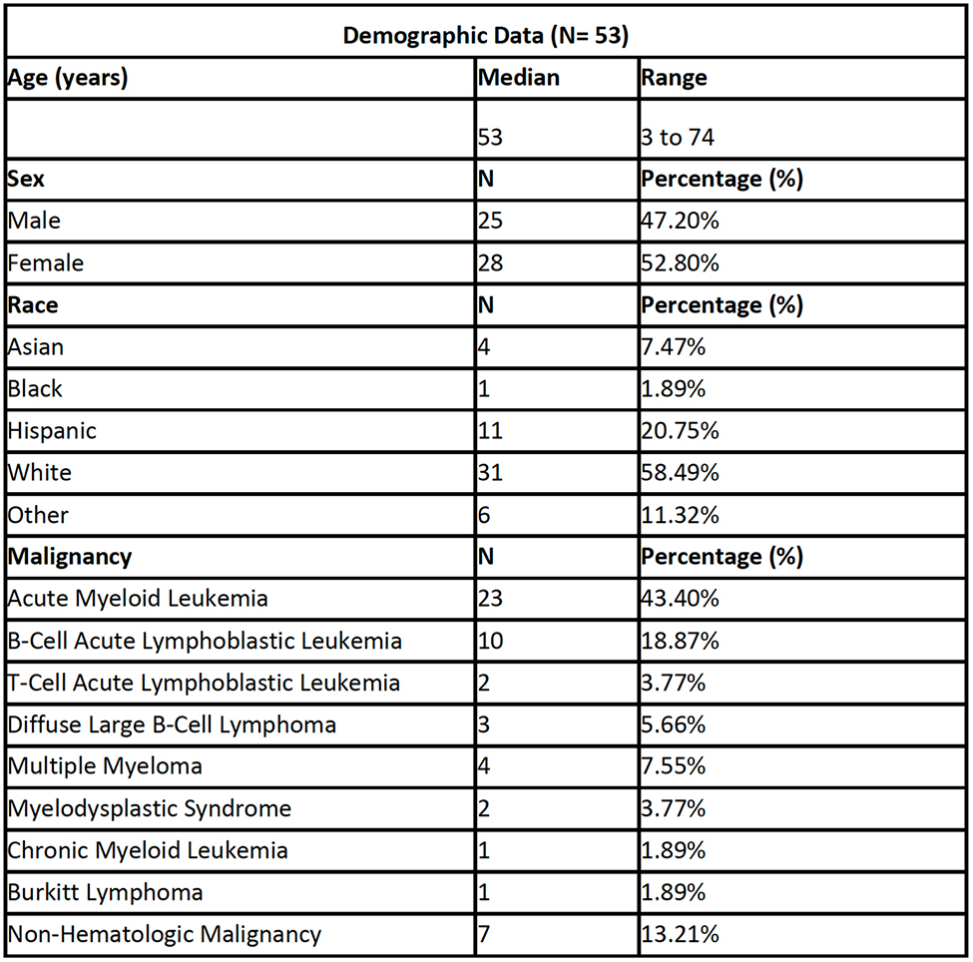
Demographic data of typhlitis patients.

**Table 2:**
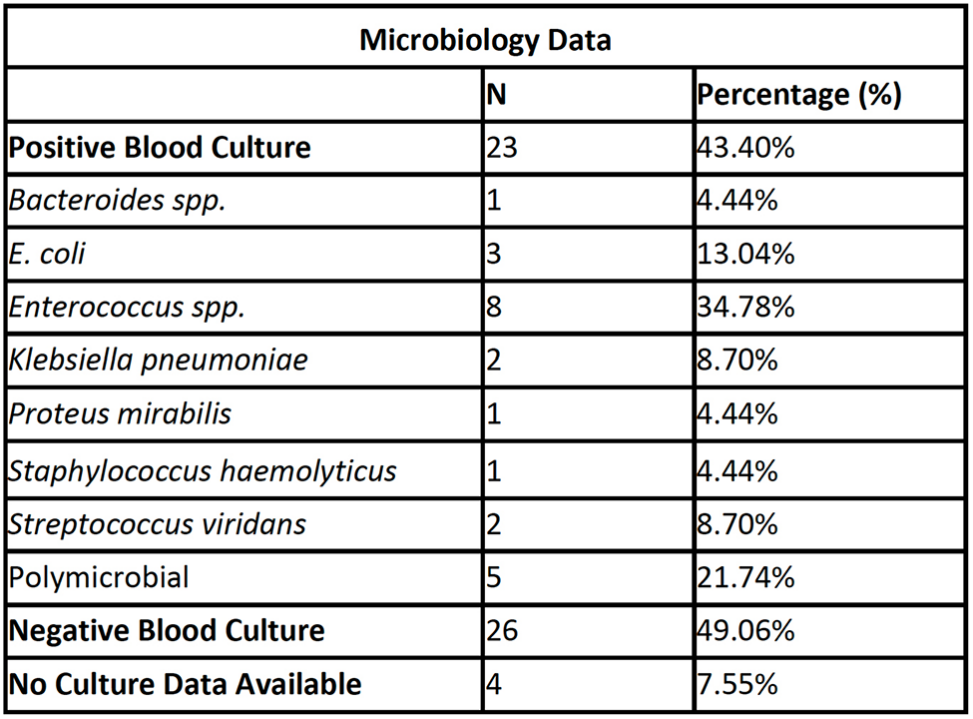
Microbiology data of typhlitis cases.

**Methods:**

A retrospective chart review was performed to identify oncology patients diagnosed with typhlitis at UC-Davis Medical Center from 2015 to 2022. Abstracted data included diagnoses, clinical and microbiology results, antimicrobial use, any resultant delays in subsequent chemotherapy, and mortality. Neutropenia severity was further characterized using the previously published D-index. Type and duration of antibiotic use was used to calculate antibiotic spectrum index (ASI).

**Results:**

53 patients (62 episodes of typhlitis) were included in the analysis (M=25, F=28). The median age was 53 years and 87% of patients had hematologic malignancy. Nine patients had two diagnosed episodes of typhlitis. In-hospital mortality was 21% and all-cause mortality was 60% at the most recent follow up. The median D-index in survivors was 5,780 compared to 9,310 in deceased (p=0.357) and median ASI was 8.727 in survivors and 8.238 in deceased (p=0.453).

**Conclusion:**

This is one of the largest case series on typhlitis to date. The attributable mortality of typhlitis was high in our cohort. Our study incorporated the novel scoring systems D-index and ASI to predict typhlitis outcomes and mortality. While these did not differ based on survival, this may have been related to the small sample size. Further areas of investigation will include comparison of typhlitis cases to matched controls without typhlitis and the application of D-index and ASI to determine if these predict development of typhlitis. Additionally, analyzing gastrointestinal microbiota in cancer patients with and without typhlitis may provide more information on the pathogenesis of typhlitis.

**Disclosures:**

George R. Thompson III, MD, Astellas: Advisor/Consultant|Astellas: Grant/Research Support|Basilea: Advisor/Consultant|Basilea: Grant/Research Support|Cidara: Advisor/Consultant|Cidara: Grant/Research Support|F2G: Advisor/Consultant|F2G: Grant/Research Support|GSK: Advisor/Consultant|GSK: Grant/Research Support|Melinta: Advisor/Consultant|Melinta: Grant/Research Support|Mundipharma: Advisor/Consultant|Mundipharma: Grant/Research Support|Scynexis: Advisor/Consultant|Scynexis: Grant/Research Support Brian Jonas, MD, PhD, AbbVie: Advisor/Consultant|AbbVie: Grant/Research Support|Amgen: Grant/Research Support|Aptose: Grant/Research Support|AROG: Grant/Research Support|Biomea: Grant/Research Support|BMS: Advisor/Consultant|BMS: Grant/Research Support|Celgene: Grant/Research Support|Forma: Grant/Research Support|Forty-Seven: Grant/Research Support|Genentech/Roche: Grant/Research Support|Gilead: Advisor/Consultant|Gilead: Grant/Research Support|Glycomimetics: Grant/Research Support|Hanmi: Grant/Research Support|Immune-Onc: Grant/Research Support|Jazz: Grant/Research Support|Kura: Advisor/Consultant|Kymera: Grant/Research Support|Loxo: Grant/Research Support|Pfizer: Grant/Research Support|Pharmacyclics: Grant/Research Support|Schrodinger: Advisor/Consultant|Syndax: Advisor/Consultant|Treadwell: Advisor/Consultant|Treadwell: Grant/Research Support

